# Classroom to crisis: Exploring nursing students’ preparedness for emergency unit placements in the Western Cape

**DOI:** 10.4102/curationis.v49i1.2813

**Published:** 2026-05-07

**Authors:** Felicity Daniels, Nomahlubi Sipamla

**Affiliations:** 1 School of Nursing, Faculty of Community and Health Sciences, University of the Western Cape, Cape Town, South Africa

**Keywords:** Clinical placement, emergency care, emergency units, nursing education, readiness, work integrated learning

## Abstract

**Background:**

Emergency nursing is complex, fast-paced, often unpredictable and focuses on the management of acute medical conditions or injured patients of all ages. It involves rapid assessment, prioritisation, timely interventions, resuscitation and stabilisation of the patient to reduce morbidity and mortality. This requires nurses to gain specific emergency care knowledge and skills.

**Objectives:**

The study investigated the experiences of student nurses in a Bachelor of Nursing programme regarding their theoretical and clinical preparation and readiness for placement in emergency units.

**Method:**

Qualitative, exploratory-descriptive methods were used. Thirty-four student nurses from a university in the Western Cape were purposively selected and participated in focus group discussions. Inductive thematic analysis was used.

**Results:**

Four themes and twelve categories were generated. The findings indicated that student nurses were inadequately prepared both theoretically and clinically for practice in emergency units. Several factors contributed to the planning and implementation challenges, leading to students’ lack of preparedness.

**Conclusion:**

Despite changes in the requirements of the South African Nursing Council for clinical placement of undergraduate students in emergency units, work integrated learning in these units would benefit graduate nurses intending to further their studies in critical and emergency care nursing. However, learning outcomes are required to ensure that student nurses have access to specific emergency care knowledge and clinical skills.

**Contribution:**

Highlights student nurses’ need for orientation, clinical supervision and debriefing sessions by competent critical and emergency care nurses, and professional counselling when needed.

## Introduction

Emergency and critical care is core to a hospital’s health care provision and plays a pivotal role, serving as a primary entry point into the health care system. This includes trauma, medical and surgical emergency and intensive care units (ICUs). Often, emergency units act as a triage mechanism for hospital admissions (Botes & Langley 2016; Kim et al. 2021).

The World Health Organization (WHO) identified traumatic injuries as a significant global contributor to mortality, with an estimated 90% of these incidents occurring in low- and middle-income countries (Edem et al. 2019; Manwana et al. 2018; Van de Ruit, Lahri & Wallis 2020). Traumatic injuries represent over 4.8 million deaths worldwide annually, leaving many survivors with long-term disabilities, which is a major public health issue (Lourens, Parker & Hodkinson 2020). Trauma causes approximately one in every 10 deaths worldwide (Oyeniyi et al. 2017). In the United States, traumatic injuries are the leading cause of death among individuals under 45 years of age (DiMaggio et al. 2016; Ibrahim et al. 2015).

A large number of patients visit public hospital emergency departments in South Africa (SA) because of traumatic injuries (Aspelund et al. 2019). The incidence of such injuries in SA is approximately seven times higher than the global average and contributes significantly to overall disease burden (Chu et al. 2022). Traumatic injuries impose significant strain on an already overburdened healthcare system because of the associated expenses of hospitalisation, diagnostic procedures, surgical interventions and with financial implications for national healthcare expenditure (Da Costa et al. 2019; Marle & Mash 2021).

Effective emergency care delivery relies heavily on an interdisciplinary team of doctors, nurses, radiologists, physiotherapists and other healthcare professionals to improve clinical outcomes for patients receiving emergency care (Seale & De Villers 2015). Nurses are recognised as the first-line healthcare professionals to engage with patients in emergency units, especially patients presenting with acute illness or traumatic injuries (Sipamla 2022). Phukubye, Mbombi and Mothiba (2021) reported that the clinical competence of emergency nurses is fundamental to ensuring high-quality emergency care. These practitioners are expected to have specialised knowledge, skills and expertise and be technically savvy to operate specialised equipment within the emergency unit (Bell et al. 2014; Dulandas & Brysiewicz 2018; Husebø & Olsen 2019; Jones, Shaban & Creedy 2015). However, extant literature highlights a lack of formal emergency care training among many nurses working in emergency units, which may negatively affect the standard of care delivered in these high-pressure environments (Dulandas & Brysiewicz 2018; Jamshidi et al. 2021).

Bayes and Ewens (2016) therefore suggest that student nurses require clinical exposure in emergency units before they qualify to ensure that they understand the unit’s dynamics. They suggest a review of nursing education institutions’ curricula to equip student nurses with relevant competencies. They further highlight the need for support services, orientation and induction and continuous professional development to prevent the stress that nurses face in this setting (Mehlape & Matlala 2024). Contrastively, Jiang, Ma and Koo (2025) caution that allowing students to practise on critically ill patients raises several ethical and safety concerns given the increasing emphasis on patient safety. This hampers the development of clinical competence and the accumulation of clinical experience in emergency care settings. They suggest that nursing education implement an innovative teaching approach that mimics real-life situations, through simulation-based education (Jiang et al. 2025).

Ekstedt, Lindblad and Lofmark (2019) argue that the structure and delivery of clinical education significantly impact the quality of students’ learning experience, which can either enhance or hinder students’ professional development. The effectiveness of clinical learning is closely associated with a supportive clinical environment and competent supervision (Memarian, Baraz & Vanaki 2015). Moreover, cultivating constructive and encouraging relationships within the clinical setting contributes to students’ ability to meet learning outcomes and fosters the development of professional attributes such as accountability, responsibility and autonomy in nursing practice (Wagoro & Rakuom 2015). In this context, an emergency unit represents a highly dynamic and multifaceted setting that offers valuable educational opportunities although it may also present challenges that affect learning outcomes positively or negatively (Afaya et al. 2021; Atakro et al. 2019).

The South African Nursing Council (SANC), a regulatory body initially established in terms of *the Nursing Act No. 45 of 1944*, currently operates in terms of *the Nursing Act No. 33 of 2005*. South African Nursing Council’s responsibilities include, among others, the provision of qualification frameworks for the development of nursing programmes according to current regulations (SANC 2020). Nursing colleges and universities in SA offer postgraduate diploma programmes in critical care nursing (child), critical care nursing (adult) and emergency nursing for those nurses who hold the requisite undergraduate qualifications and who wish to specialise in these fields (SANC 2010). According to the SANC qualification framework and programme completion document for students registered for the undergraduate programme and as per Regulation 425 (R425) (recently phased out), students must have had a work-integrated learning placement in the casualty ward, out-patient department and operating theatre (SANC n.d.). However, there were no clearly defined standards, guidelines and learning outcomes for work-integrated learning in these units for the R425 programme (Sipamla 2022).

Regulation 174 (R174) does not stipulate these work-integrated learning requirements for the new Bachelor of Nursing programme. However, student nurses must be competent in basic life support in maternal and neonatal resuscitation (SANC 2025). Exposure to emergency units may stir graduates’ interest in postgraduate studies in critical care or emergency nursing. Prior exposure might also prove beneficial during postgraduate studies, as well as for practice in these units after obtaining the postgraduate qualification. This is particularly important considering that a study done in Gauteng (SA) reported that newly qualified professional nurses are often assigned to work in ICUs because of a severe shortage of trained staff (Mehlape & Matlala 2024). However, minimal exposure to this environment results in a lack of the required knowledge, resulting in poor clinical judgement and decision-making by unqualified critical care and emergency nurses. In addition, students’ readiness to work in emergency units was put to test during the coronavirus disease 2019 (COVID-19) pandemic when a severe staff shortage in high care and ICUs resulted in the placement of final-year student nurses in these units in some provinces in SA (Olivier & Downing 2025). These authors studied registered final-year bridging course (Regulation 683) student nurses at a private nursing education institution in SA and documented that although students practise some skills in simulation during their basic training, they did not feel competent to perform these skills in the emergency units (Olivier & Downing 2025).

The lack of clear learning outcomes to guide what undergraduate student nurses should learn in emergency units may, therefore, negatively impact the development of emergency care competencies and ultimately patient outcomes (Sipamla 2022).

### Aim

The study investigated the experiences of student nurses in a Bachelor of Nursing programme regarding their theoretical and clinical preparation and their readiness for placement in emergency units (Sipamla 2022).

## Research methods and design

The current study adopted a qualitative research approach and an exploratory descriptive design. It employed Pamela Baxter’s Communication, Collaboration, Application, Reflection and Evaluation (CCARE) model as the conceptual framework to bridge the gap between theory and practice (Sipamla 2022; Zyoud 2023). The key behaviours outlined in the CCARE model for linking theory to practice include communication, collaboration, application, reflection and evaluation. The model emphasises that collaboration and coordination between the students, the preceptor – clinical facilitator based at the clinical facility, and the clinical facilitator or nurse educator – based at nursing education institution – university or college, are essential for linking theory to the practice of patient-centred care. A preceptor plays the most crucial role; therefore, regular meetings with students to set goals, share policies and obtain feedback are important. The model recognises that student and preceptor interactions, whether positive or negative, impact a student considerably. During evaluation, student performance is assessed, and constructive feedback makes students aware of their performance and the theory and practice links (Zyoud 2023). The CCARE model was applied in the study, considering the vital role of the clinical staff as preceptors in emergency care units and the role of the clinical facilitators as clinical educators, who serve as important liaisons between education and practice (Sipamla 2022).

### Research setting

A higher education institution in the Western Cape served as the current research setting. At this institution, students are placed at community and hospital emergency units for work-integrated learning and are supervised by clinical facilitators employed by the university. In the absence of the clinical facilitators, students work under the supervision of staff employed at the clinical facility (Sipamla 2022).

### Population and sampling

Registered Bachelor of Nursing third- and fourth-year student nurses at the Western Cape higher education institution made up the study population. A previous clinical placement in a medical, surgical or trauma emergency unit was an inclusion criterion for participation in this study. Participants were recruited after class sessions at the university. Thirty-four participants met the inclusion criteria and were sampled through non-probability, purposive sampling (Sipamla 2022).

### Data collection instrument

A focus group interview guide was developed using extant literature to facilitate data collection. The interview questions focused on experiences of students’ theoretical and clinical preparedness and readiness for clinical placement in emergency units (Sipamla 2022). The following were posed to participants and explored using probes:

- *‘Share your experience of the theoretical and clinical preparation you had for clinical placements in emergency units.’*
- *‘What are your perceptions about your theoretical and clinical readiness for placements in emergency units?’*

### Data collection process

Data were collected by the researcher, a Master’s Degree student, in a quiet venue at the university. The study was explained to potential participants. Their rights to voluntary participation, informed consent and the right to withdraw from the study were also explained. Consent and focus group binding forms were completed and participants consented to the use of an audio recorder. Participants were addressed using unique numbers to ensure anonymity. The focus group discussions were conducted in English, audio recorded and lasted from 45 min to 1 h. Data saturation was done after five focus group discussions (Sipamla 2022).

### Ethical considerations

Ethics approval and clearance were granted by the Humanities and Social Science Research Ethics Committee (HSSREC) Reference: HS20/9/36. The Registrar granted permission for the study to be conducted at the university. Participation was voluntary and with participant’s informed written consent. Participants were assured of anonymity and confidentiality and could withdraw at any time without penalty. A data management plan, drawn up according to university guidelines, was followed to ensure adherence to *the Protection of Personal Information Act* (Sipamla 2022).

### Data analysis

Data were analysed manually following the inductive, thematic approach proposed by Creswell (2014). The audio-recording was listened to and then transcribed verbatim by the researchers. The transcripts were read thoroughly for sense-making. A significant number of codes were identified during this process before grouping them together, based on emerging patterns, to generate categories. Four themes and 12 categories were generated from the data. The research supervisor reviewed and confirmed the themes and categories. All transcripts and audio-recordings were shared with the research supervisor for confirmation of the themes (Sipamla 2022).

### Trustworthiness

Safeguarding credibility, dependability, transferability and confirmability (Lincoln & Guba 1985) ensured trustworthiness of the present study. The researcher conducted a mock interview to ensure that the interviewing process and technique were effective and enhanced credibility. Audio-recordings and field notes were preserved safely to ensure collected data’s accuracy. The audio-recordings and transcripts were made available for checking by the participants to ensure accuracy and authenticity. Detailed descriptions of the research participants, setting and process were provided; an audit trail was maintained to establish data transferability to other contexts. Confirmability with the research process was ensured through researcher reflexivity and constant discussions with the research supervisor (Sipamla 2022).

## Results

Eighteen nursing students in the fourth year of study, 16 females and 2 males, and 16 students in the third year of study, 9 females and 7 males, participated in the study. Nineteen of the 34 participants had a work-integrated learning placement in community healthcare centre emergency units, and 15 in hospital emergency units. The 4 themes and 12 categories are supported by direct participant quotes which are referenced using a participant number, their year of study and gender.

## Discussion

### Theme 1: Emergency-based education is insufficient

This theme focuses on students’ experiences of their theoretical and clinical education on emergency nursing. According to the CCARE model, effective collaboration and coordination between the preceptors, clinical supervisors and students regarding students’ learning needs could have been prevented.

#### Category 1: Exposure to simulation of clinical skills in the laboratory was insufficient

The students were only exposed to checking an emergency trolley, which is a skill linked to emergency nursing. According to students, this was insufficient for application in emergency situations such as resuscitation. Students asserted:

‘… I did not even know some of the equipment. I know there is an emergency trolley and it is where I am supposed to get things. But I did not know how to connect things; how to and what to do to help them.’ (P3, Yr4, M)

‘So, the only thing that we were prepared for was the emergency trolley of which it was only done like in skills lab. What do you get under first drawer, second drawer, third drawer and some other equipment, you are told how to use them. But you are not really exposed on how to use them on real patients or real [*humans*].’ (P1, Yr3, M)

‘I feel like they do n’t teach us basics like triaging a patient and nebulising a patient, or putting in a drip. We must learn all those things by ourselves.’ (P6, Yr3, F)

Citolino et al. (2015) conducted a study in Brazil on factors affecting the quality of cardiopulmonary resuscitation (CPR) in inpatient units. They also identified that participants were not familiar with the emergency trolley (98.0%) and lacked harmony during CPR. Although nurses are responsible for checking and restocking the emergency trolley, they have insufficient knowledge and practice in this regard (Kaushik 2019).

Salifu, Heymans and Christmas (2022) conducted a study in Ghana on the exploration of nursing students’ experience and perception in the teaching and learning of clinical competencies and found non-prioritisation of practical skills. This is a concern, as patients who are admitted to emergency units are at high risk of acute deterioration and death (Bogossian et al. 2013). Considine et al. (2021) observed an increase in mortality in emergency units when deterioration in the patient’s condition is not identified and responded to promptly.

Skills laboratories are safe spaces for student nurses to be prepared to recognise and intervene when a patient’s condition worsens and to prevent patient death. The use of immersive technologies such as virtual reality (VR) and augmented reality (AR) makes clinical education more life-like (Sipamla 2022). Together with training in basic life support, the use of these technologies in clinical education prepares student nurses for the real world, for example, the training of students in triaging, to initially assess and prioritise patients using the South African Triage Score.

#### Category 2: Clinical skills taught in the skills laboratory are performed differently in practice

The discrepancy between the teaching of clinical skills and the practice of skills in the emergency unit was highlighted. A student explained:

‘… And when we got to facilities to practice … it’s totally different (…) the way they manage the patient.’ (P4, Yr4, F)

However, such discrepancies should be used as discussion points between students, clinical facilitators and emergency nurses to develop students’ critical thinking and clinical reasoning.

Kertu and Nuuyoma (2019) conducted a study among student nurses and found an inconsistency between theory and clinical practice, which caused confusion and discouragement. Hashemiparast, Negarandeh and Theofanidis (2019) argue that performing clinical skills in a skills laboratory is different from when performing it in clinical practice. Some emergency clinical skills, according to Kalyani et al. (2019), are not easy to simulate. Hence, Svellingen et al. (2021) suggest integration of simulation and practice (Sipamla 2022).

Curricula must therefore be reviewed to establish the effectiveness of clinical learning and the integration of knowledge and skills (Abu Salah, Aljerjawy & Salama 2018). This allows for the alignment of learning and teaching to evolving nursing practice (Fawaz, Hamdan-Mansour & Tassi 2018). Collaboration between higher education institutions and speciality clinical areas is, however, essential to ensure the smooth integration of theory to practice.

#### Category 3: Theoretical preparation is insufficient for practice in emergency units

Students were dissatisfied with their preparation around the specific learning outcomes and prerequisite knowledge required for placement in the emergency units. They were unclear about their roles and duties in the emergency unit (Sipamla 2022). This was expressed as follows:

‘Okay, I feel like a lot of what I learned in trauma, I learned it from the facilities themselves. I didn’t learn it here at the university.’ (P1, Yr3, M)

‘… There is no specific preparation for a trauma unit placement.’ (P7, Yr4, F)

‘In theory, what happens is they will just like say, okay, this is the guidelines of emergency, but they won’t go into detail.’ (P4, Yr4, F)

Studies by Dulandas and Brysiewicz (2018) in KwaZulu-Natal and by Aloyce, Leshabar and Brysiewicz (2014) in Dar es Salaam revealed that most nurses working in emergency units lack formal training in emergency nursing. The lack of training, according to Aloyce et al. (2014), affects clinical judgement and threatens effective emergency management, which increases the risk of disability and mortality. According to Hynus and Karama (2017), formal and structured emergency nursing guidelines and a well-trained workforce are needed to deliver quality emergency care (Sipamla 2022). According to the CCARE model, effective collaboration and feedback between the preceptors, clinical facilitators and students regarding students’ learning needs and the gaps could have prevented students’ negative experiences (Zyoud 2023).

### Theme 2: Educational planning challenges

Students’ experience of the planning and implementation challenges related to their clinical placement in trauma, surgical and medical emergency units is the focus of Theme 2.

#### Category 1: Lack of synchrony between theory and work-integrated learning

The lack of synchrony between theory and practice was highlighted. Lectures on emergency nursing did not coincide with simulation in the skills laboratory and placement for work-integrated learning. Students opined that they also had limited exposure to emergency units and did not rotate to all types of emergency units, resulting in lost learning opportunities. A student reported:

‘So, I think in terms of placement, the theory and the placement periods don’t match up. So, which means we are placed, say for example, in first semester, we are placed in trauma or theatre. But only in second semester, we are taught theory on trauma … it doesn’t match up.’ (P9, Yr4, F)

Kalyani et al. (2019) found that inadequate educational planning led to difficulties in the clinical setting, particularly in terms of insufficient preparedness and diminished self-confidence. Similarly, Safazadeh et al. (2018) highlighted that ineffective planning in education often results in students engaging primarily in routine tasks within emergency units. Aligning students’ clinical experiences with theoretical preparation is therefore critical to facilitate effective learning (Saifan et al. 2021).

#### Category 2: Communication challenges between nursing education institution and practice

Students reported a lack of consistency in communication between the nursing school and the healthcare facility where they undertook work-integrated learning. They indicated that the breakdown in communication negatively impacted their learning experiences in emergency units. The absence of clearly defined roles and learning outcomes led to inadequate supervision and guidance from clinical nursing staff. Furthermore, students felt apprehensive about expressing their learning needs within the clinical setting. They believed that improved communication between the school of nursing, clinical facilities and students could boost their confidence and motivation to engage in learning within emergency units (Sipamla 2022). Students reported:

‘So, they [*School of Nursing*] expect the facilities to teach us what we don’t know. But then the facilities also expect us to know, so there are expectations from the school and the clinical that we are not meeting, so there’s that gap.’ (P4, Yr4, F)

‘I think what could help now is the hospitals or the facilities, keeping up with the universities in terms of the objectives …, because the RNs already [*registered nurses*] know what is supposed to be done by students. Many students are scared to voice out their objectives.’ (P7, Yr4, F)

This finding corroborates results by Jahanpour et al. (2016) on nursing students in Iran and by Fadana and Vember (2021) in the Western Cape, which found that poor communication between nurse educators, clinical nurses and student nurses negatively impacted students’ clinical learning experience (Sipamla 2022). Mamaghani et al. (2018) similarly identified issues of educational ambiguity among participants in Iran, which contributed to confusion in the learning process. Such challenges have been linked to a range of negative emotional responses among student nurses, including feelings of vulnerability, anxiety, worry, decreased confidence and motivation, a sense of isolation and a lack of preparedness during clinical training (Atakro et al. 2019; Farzi, Shahriari & Farzi 2018). Jamshidi et al. (2016) suggest that proper communication increases students’ motivation. According to the CCARE model, collaboration is a key behaviour for linking theory to practice (Zyoud 2023). Evidently, collaboration between the clinical facilitators, preceptors and students, regarding the synchrony of theory and practice and learning outcomes for work-integrated learning, is crucial to improve students’ experiences.

### Theme 3: Students’ lack of readiness for work-integrated learning in emergency units

This theme focuses on students’ perceptions of their theoretical and clinical readiness for work-integrated learning in emergency units.

#### Category 1: Knowledge and skills were inadequate

Students reported insufficient theoretical knowledge and exposure to clinical competencies prior to their placement in emergency units for work-integrated learning. They highlighted limited exposure to emergency units and noted a disparity between their expectations and the actual demands encountered in emergency units. According to the students, the lack of preparedness hindered their readiness for clinical practice in emergency units. This contrasts with the view of Althiga et al. (2017), who assert that student nurses should have a solid theoretical foundation, essential clinical skills, effective communication, cultural competence, professional values and ethical behaviour before clinical placement (Sipamla 2022). The following were students’ assertions:

‘So, we haven’t even had any theory. Well, we had some in second and in first year, but it wasn’t really trauma-based or emergency setting-based. So, I would say in my opinion, I was not ready.’ (P8, Yr3, M)

‘But from my experience, I wasn’t that exposed to a lot of trauma or emergency cases or medical emergencies. So, I would definitely need some more experience to find my feet in an emergency ward.’ (P3, Yr4, M)

‘But they don’t really prepare you for the role that you will have when you are there at the adult resuscitation itself.’ (P1, Yr3, M)

These findings support those of Wahyuningsih et al. (2020), who found that 77.3% of student nurses felt unprepared to manage emergency situations because of limitations in their education and clinical experience. Similarly, Memarian et al. (2015), in a study involving nursing students in Iran, identified several barriers to effective clinical learning, including insufficient theoretical knowledge, inadequate clinical preparation and a lack of proper clinical supervision (Sipamla 2022).

#### Category 2: Students were not emotionally prepared

Majority of the students reported experiencing emotional distress during their clinical placements, primarily because of unexpected exposure to traumatic events for which they felt unprepared. Consequently, many indicated a need for emotional support as they found that supportive resources within the clinical environment were insufficient. Students perceived that such traumatic experiences often elicited a range of negative emotional responses, including fear, anxiety and sadness (Sipamla 2022). The following was shared:

‘Yes, some of them come with … gunshot wounds and stuff. So … we are … scared, to fully participate in those things.’ (P4, Yr4, F)

‘I was working at a hospital … a children’s hospital. So, a five-year-old was raped and she was bleeding on the mattress. So, on my side, it is not something I can stand with … when I see someone comes with like a gunshot wound, seeing like the blood is oozing like water from a pipe, it is not something I can see like, in front of me all the time.’ (P3, Yr4, M)

‘No theory comes and tells you that a gunshot is going to be on the arm or on the head. The events are too traumatic. And I don’t think or I don’t remember … if there was ever a class where you are told how to be prepared, mentally prepared to deal with the traumatic events that present in the trauma unit.’ (P8, Yr3, M)

According to Sipamla (2022), students experienced psychological trauma as a result of their exposure to critical incidents in the trauma unit. These findings are supported by Motsaanaka, Makhene and Ally (2020), whose study on student nurses’ clinical placement experiences in Gauteng revealed that students frequently encountered negative emotional responses. Emotional distress among student nurses has also been linked to their strong desire to save lives despite lacking the necessary clinical experience (Getiérrez-Puertas et al. 2021). Similarly, Jiménez-Herrera et al. (2020) reported in Spain that emergency nurses often face emotional challenges when they are unable to implement best practices because of external constraints. These nurses, being morally attuned to patient vulnerability, experience a loss of control in situations where they are prevented from delivering optimal care, which in turn leads to negative emotions such as helplessness, frustration, anger and guilt (Getiérrez-Puertas et al. 2021; Jiménez-Herrera et al. 2020).

The emergency care unit is a dynamic and unpredictable environment, with a constant influx of diverse patients, which can be overwhelming for students. Although this setting can stimulate motivation to learn, it also fosters feelings of helplessness and fear of the unknown. Meng et al. (2019) argue that clinical facilitators play a critical role in reducing this psychological stress by properly orienting and supporting students. Although somewhat limited, evidence suggests that realistic simulation-based training can enhance students’ understanding, communication skills and psychological readiness to cope with complex clinical settings (Zeng et al. 2020).

#### Category 3: Feelings of anxiety and fear

Students reported experiencing fear, nervousness and a sense of helplessness while placed in emergency units. These emotional responses were primarily attributed to their anxiety about managing unpredictable and unfamiliar situations, compounded by a perceived lack of preparedness. Reflecting on their experiences, students noted that exposure to unforeseen clinical scenarios triggered negative emotions, which in turn adversely affected their ability to engage effectively in the learning process within emergency care settings (Sipamla 2022). Students mentioned the following:

‘Nervous, anxious, confused and yeah … But I don’t feel like I would be ready enough.’ (P1, Yr3, M)

‘I saw a patient coming in with a massive gun gunshot wound …blood … coming out of it … So, the first thing that you do instead of helping – you freeze … because mentally you’re not prepared to think that you’re ever going to see someone who’s losing so much blood.’ (P8, Yr3, M)

‘It was kind of frightening … it was my first time experiencing such trauma … Yeah, it was kind of scary, but I got used to it.’ (P4, Yr4, F)

Belayneh et al. (2021), in a study conducted in Ethiopia, reported that nurses employed in emergency units experienced elevated levels of anxiety. To mitigate anxiety during clinical placements, Simpson and Sawatzky (2020) recommend that nurse educators adopt proactive strategies, including providing thorough orientation, conducting pre-clinical workshops, ensuring students possess foundational knowledge and incorporating simulation-based learning. Feedback, as a means of collaboration is key, according to the CCARE model (Zyoud 2023). As proposed by the model, regular meetings and in-service training could afford the student the space to share their concerns, fears and anxiety, which could be addressed timeously by the preceptors and clinical facilitators (Sipamla 2022).

### Theme 4: Learning experiences were both positive and negative

The theme relates to the students’ learning experiences during work-integrated learning in emergency units.

#### Category 1: Students learnt by doing

Students reflected on their experiences of work-integrated learning and the informal exchange of knowledge among nursing staff. They described emergency units as dynamic, high-pressure environments characterised by complexity and unpredictability when dealing with a wide range of patient conditions. According to the students, emergency nursing demands specialised expertise and the ability to adapt quickly, think critically, prioritise care, manage complex situations and implement resuscitative interventions, all within a collaborative multidisciplinary framework. They viewed this clinical setting as offering valuable hands-on learning opportunities that not only enhanced their motivation but also provided insight into the scope and specialised nature of emergency nursing practice (Sipamla 2022). Students stated the following:

‘I said I’m scared to put up a drip. They said, Oh, okay, and then I will show you how to do it so that you know … that experience was good.’ (P8, Yr3, M)

‘They were very nice, like, how can I say, I felt very at home there. And that’s why I also learned a lot.’ (P5, Yr3, M)

‘So, then I felt a sense of, okay, now I know what to do. And when the sister saw that you were competent, that’s when they actually gave me my freedom to be here on the floor. They weren’t … they were very supportive … I felt very big, or very grown after a while.’ (P8, Yr3, M)

This is congruent with a study done in Sweden by Ewertsson et al. (2017), which reported that student nurses experience of hands-on learning is important to complement previous knowledge and increase students’ confidence. The development of clinical experience and competence is highly dependent on sufficient clinical exposure of student nurses (Sipamla 2022).

#### Category 2: Clinical supervision was inadequate

Several students reported inadequate clinical supervision during their placements. They indicated that clinical facilitators appeared primarily concerned with verifying clinical hours rather than actively engaging in clinical teaching. Facilitators often lacked clarity regarding their supervisory role within the emergency unit, which resulted in students being assigned routine tasks instead of participating in meaningful learning activities. The absence of defined roles and specific learning outcomes for the placement further hindered meaningful clinical engagement. Students emphasised that effective clinical supervision is essential for bridging the gap between theoretical knowledge and clinical application, as well as for mitigating negative emotional experiences during work integrated learning. A student commented:

‘I think our supervisors just come and check on us but they don’t teach us. They don’t give us the knowledge … for example, if you come to the hospital as a supervisor, you must tell me in this ward, now, this is what you’re supposed to be doing.’ (P6, Yr3, F)

Their experiences are aligned with the findings of a study on student nurses by Berhe and Gebretensaye (2021) in Ethiopia, which reported on clinical supervision inadequacy. Clinical supervision is one of the pillars in nursing education, which is necessary for efficient learning and teaching in clinical education (Donough & Van der Heever 2018). In this regard, clinical facilitators are the first source of student support (Zabihi, Jafarian-Amiri & Qalehsari 2020). Atakro et al. (2019) and Safazadeh et al. (2018) revealed that clinical supervision in emergency units is difficult because of some clinical facilitators being inexperienced, having insufficient knowledge and showing incompetence in working with some equipment in emergency units. This leads to student nurses expressing feelings of vulnerability, anxiety, worry, loss of confidence, motivation, isolation and unpreparedness during clinical learning (Atakro et al. 2019; Farzi et al. 2018).

#### Category 3: Lack of involvement during resuscitation

Some students reported feeling rejected during resuscitation. In their opinion, students lost the learning opportunity and teamwork with the multidisciplinary team. The following were some students’ assertions:

‘If it’s an emergency case, they will not give much attention to second year students.’ (P9, Yr4, F)

‘But especially with resuscitations, we were “shooed” away … even if you wanted to help.’ (P6, Yr3, F)

‘… maybe resuscitation is happening, by the time you turn around the resus [*resuscitation*] is done, you don’t really get to learn … maybe in trauma the most we are allowed to do is to manage an asthmatic patient because you must … give nebs [*nebulisers*] to this patient.’ (P8, Yr3, M)

This observation is contrary to findings by Roel and Bjørk (2020) and Nasr-Esfahani, Yazdannik and Mohamidiriz (2019), who argued that nursing students should be able to initiate and perform CPR when they start their nursing career. Often, the nurse arrives first at a scene of cardiac arrest in the hospital; therefore they must be competent in CPR.

Student’s rejection at the point of CPR results in loss of the opportunity to experience and perform the procedure, master the skill, transfer theory into practice and reduces interaction with the multidisciplinary team, patient and family (Memarian et al. 2015).

#### Category 4: Patient care took precedence over students’ learning needs

Students indicated they are regarded as part of the team and not a student with learning needs. Students reported that they often did routine work such as observations, transferring patients to the ward and theatre and were asked to work in other units because of a shortage of nurses. Based on their reflections, emergency units focused on patient care over student learning needs. There was limited exposure, insufficient support, guidance and supervision both from clinical facilitators and facility staff.

Some students reported:

‘What we got was you have to take blood, you have to test urine, you have to take something like HBs [*hemoglobin tests*]. It was simple things like that.’ (P1, Yr3, M)

‘You find for the five days … you go home tired, but you learned nothing.’ (P9, Yr4, F)

Using student nurses as an extra pair of hands results in inadequate exposure to learning experiences, while doing routine duties lead to frustration (Jamshidi et al. 2016). Engaging in routine-based tasks and repetitive procedures may also reduce student’s motivation to learn.

### Limitations of the study

The study was conducted only at one higher education institution; therefore, the findings of this study cannot be generalised to students in other Bachelor of Nursing programmes at other institutions and in different contexts. The study focused only on the Bachelor of Nursing students and excluded clinical facilitators, nurse educators, nurse managers and clinical nursing staff. Inclusion of these participant groups might have provided a more comprehensive understanding of the phenomena.

### Recommendations

Improved relationships between nurse educators and clinical staff are recommended to facilitate the integration and synchrony of theory and practice and facilitate maximum rotation of students through emergency units. Student supervision should not be an administrative exercise but must include bedside teaching, learning and support, acknowledging that students are not expected to be emergency nurse specialists. As far as possible, clinical facilitators supervising students in emergency units should be qualified in emergency nursing to ensure that the engagement with students goes beyond administration. More attention should be given to checking whether student’s skills registers are signed off, and that they have met the learning outcomes for the placement. Students should be introduced to first response mnemonics, for example, the ABCDE (airway, breathing, circulation, disability, exposure) and PRICE (protection, rest, ice, compression and elevation) approaches and the SA Triage Scale, which will help standardise education and practice. Case- related assessments should be encouraged to keep students motivated to learn in the emergency units. Student reflective journals should be encouraged as these are powerful tools for obtaining feedback on the student’s experiences, which can inform work-integrated learning improvement plans. Emergency nursing specialists should assist with orientation and in-service training for nursing students on emergency nursing care to prepare them for work-integrated learning and to prevent the experience of the placement as traumatic. Debriefing and student counselling should be available to students, where necessary, and opportunities to discuss their experiences in class should be created. These recommendations might require further curriculum reforms specifically for undergraduate nursing programmes.

## Conclusion

We can conclude that clinical placement in emergency units can provide student nurses with a positive or a negative clinical learning experience. Their experiences and readiness for work-integrated learning in emergency units depends on specific emergency knowledge, skills, communication, support and sufficient clinical supervision. The study provides evidence that the Bachelor of Nursing students were not ready for clinical placements in emergency units. Challenges with planning of theoretical and clinical education in emergency care resulted in students’ lack of development of the required competences. Overall, it is recommended to strengthen systemic adjustments in both education and practice to improve student’s emergency care readiness to facilitate a positive contribution to patient outcomes.
